# MedAssist: LLM-Empowered Medical Assistant for Assisting the Scrutinization and Comprehension of Electronic Health Records

**DOI:** 10.1145/3701716.3715186

**Published:** 2025-05-23

**Authors:** Ran Xu, Wenqi Shi, Jonathan Wang, Jasmine Zhou, Carl Yang

**Affiliations:** Emory University, Atlanta, Georgia, USA; UT Southwestern Medical Center, Dallas, Texas, USA; Emory University, Atlanta, Georgia, USA; Emory University, Atlanta, Georgia, USA; Emory University, Atlanta, Georgia, USA

**Keywords:** Electronic Health Records, Retrieval, Large Language Models

## Abstract

Efficiently comprehending diagnosis and treatment plans remains a significant challenge for both medical professionals and patients, particularly when dealing with rare or newly emerging diseases and specific combinations of comorbidities. We present MedAssist, a large language model (LLM)-empowered medical assistant designed to support the scrutinization and comprehension of electronic health records (EHRs). MedAssist leverages two key components: *medical knowledge retrieval*, which retrieves the latest and most comprehensive medical knowledge snippets from the web, and *data retrieval*, which extracts diagnosis and treatment plans for similar patients from existing EHR databases. By integrating these capabilities into user-friendly interfaces, MedAssist bridges critical gaps in medical knowledge accessibility and understanding, and advances patient care in realistic clinical scenarios.

## 1 Introduction

Electronic Health Records (EHRs) are systematically collected across diverse healthcare institutions, covering comprehensive patient information such as diagnoses, medications, and laboratory results. In clinical research and practice, clinicians utilize EHR systems to access relevant cohort data, spanning detailed individual records to population-level insights, to support clinical decision-making and improve the quality and efficiency of healthcare delivery [2].

However, generating comprehensive diagnoses and treatment plans remains a significant challenge due to the reliance on extensive data engineering support to extract information from EHR systems and external knowledge bases. These inefficiencies highlight the need for an automated EHR assistant system capable of streamlining data analysis and improving clinical accuracy, thereby optimizing the efficiency and effectiveness of EHR workflows.

Large language models (LLMs) [5] brings us one step closer to achieving such automated EHR assistants, as with strong text encoding [9, 15], instruction-following [6], and reasoning abilities [7]. These properties enable LLMs to bridge the gap between the complexity of EHR data and actionable insights, facilitating tasks such as extracting relevant information, contextualizing medical codes, and generating personalized patient summaries. Although recent studies have explored adapting LLMs to EHRs, they mainly focus on target disease prediction [12], text-based question answering [3, 13] or data analysis [10]. However, there is a notable lack of a unified framework for systematically integrating LLMs into broader EHR workflows to support clinical decision making.

### Research Project.

In this project, we develop MedAssist, a user-guided system designed to help users scrutinize and comprehend diagnoses and treatments, thereby improving patient care. MedAssist features user-friendly interfaces tailored for both clinicians and patients and integrates two key modules: (1) *knowledge retrieval from external sources*, which retrieves relevant literature and knowledge bases to augment patient data. This step provides contextual insights into medical codes. (2) *data retrieval from internal records*, which effectively processes complex user queries to extract and reason over query-specific medical information from existing EHR databases. By integrating external knowledge with local EHR data, MedAssist generates informative and useful summaries to improve patient understanding as well as support clinical decision-making.

### Fit with the WWW ecosystem.

This demonstration is highly relevant to researchers in Data Mining, Information Retrieval, and Health Informatics, as it showcases the integration of advanced language models with web-based medical data to enhance information retrieval and comprehension. The system leverages web technologies to process and summarize extensive medical literature and EHRs, facilitating improved understanding and decision-making in healthcare. By addressing challenges in accessing and interpreting web-based medical information, MedAssist aligns with the conference’s focus on innovative web applications and their societal impact. The core methods of the system will be open-sourced, allowing users to customize them within their local clinical environments.

## 2 The MedAssist Framework

**Figure 2:**
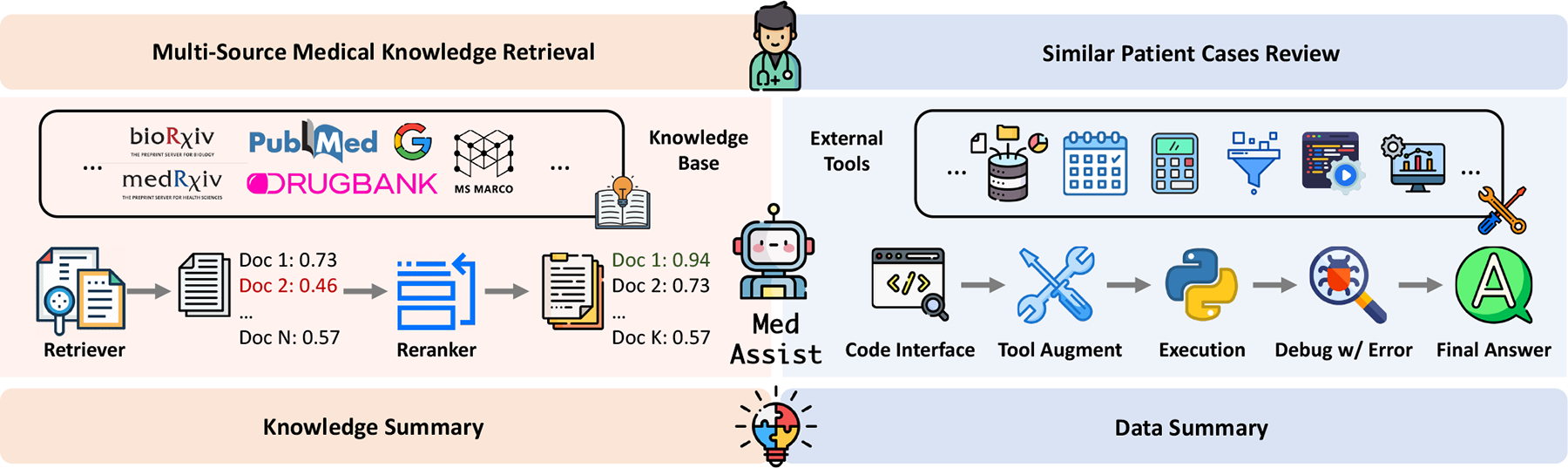
Overview of MedAssist. It incorporates retrieval to extract customized information from *external medical knowledge sources* and *internal health records*. This customized context is then utilized to generate personalized patient summaries.

In this section, we present a detailed description of the MedAssist framework. As depicted in Figure 2, MedAssist encompasses several key steps: (1) *knowledge retrieval from external resources*: it collects diverse knowledge resources, converts them into text format, and uses user input to retrieve tailored knowledge from external sources based on specific queries. (2) *data retrieval from internal records*: it decomposes complex queries into a sequence of manageable actions, utilizing external toolsets to navigate EHRs. It facilitates seamless clinician interaction with EHRs using natural language. (3) *patient summary generation*: it integrates local EHR data with LLMs to produce concise and informative patient summaries, combining insights from both internal and external knowledge sources.

### 2.1 Knowledge Retrieval from Medical Corpora

Retrieval serves as an effective tool to inject external knowledge to existing models without expensive parameter update, and have been applied to health domain with success [14, 16]. To leverage the semantic richness of medical codes for enhanced summarization, we retrieve relevant knowledge for each medical code (e.g., diseases, symptoms, medications) in EHRs using its surface name. Intending to ensure comprehensive clinical knowledge coverage, we curate a diverse external corpus comprising PubMed, DrugBank, MeSH, Wikipedia, and MS MARCO. Each knowledge unit is represented as raw text to enable efficient retrieval. For the retrieval process, we employ our developed BMRetriever [15], which archives state-of-the-art performance on a broad suite of biomedical retrieval tasks and can follow human instructions for retrieval well.

Specifically, we first use BMRetriever *R*(·) to build an index for corpus ℳ to support retrieval. At runtime, we map each medical code *q*, paired with a user-defined instruction *I* (e.g., specifying the type of information required), into an embedding vector aligned with the corpus passage embeddings. The similarity between the query and a passage *d* is computed as: *f* (*I, q, d*) = *R*[*I*, *q*])^⊤^*R*(*d*). For the medical code *c*_*i*_ with the surface name *s*_*i*_, we retrieve top-*N* passages 𝒯_*i*_ from the corpus ℳ as

(1) $${𝒯}_{i}=\underset{d\in ℳ}{\text{Top}-k}\;f\left(I_{i},s_{i},d\right).$$

To improve the quality of retrieval results, ranking often serves as a intermediate step to filter out low-quality passages [17]. We enhance this process by reranking the top-*N* retrieved passages using a pre-trained cross-encoder [1]^1^. Specifically, for each passage *d*_*i*_ among retrieved *k* passages, we concatenate medical code *c*_*i*_ and passage p as the input of the pre-trained cross-encoder. For each input, it will output a scalar value between 0 to 1, which is then used as reranking scores for *k* passages. The top-ranked passages are considered as the external knowledge 𝒦_*i*_ for the medical code *c*_*i*_. In our system, we set *N* = 25*, k* = 5 to balance between retrieval accuracy and inference latency.

### 2.2 Data Retrieval from Internal Records

To address complex user queries that require extracting information from internal EHR databases, MedAssist leverages EHRAgent [8] for multi-turn interactive coding with external tools, enabling multi-hop reasoning. We incorporate query-specific medical information for effective reasoning based on the given query, guiding MedAssist to identify and retrieve the relevant tables and records with few-shot examples. To enable LLMs in complex operations such as calculations and information retrieval, MedAssist integrates various external tools for EHR interaction, detailed as follows:

- *Database Loader*: It loads a specific table from the database.
- *Data Filter*: It filters the loaded table based on conditions defined by a column name and a relational operator (e.g., “<“ or “>“).
- *Get Value*: It retrieves all values from a specific column or performs basic operations (e.g., mean, max, min, sum) on those values.
- *Calculator*: It performs calculations from input strings using the WolframAlpha API^2^. It supports both simple operations (e.g., addition, subtraction, multiplication) and more complex ones (e.g., averages, maximum values).
- *Calendar*: It computes the date based on an input and time interval.
- *SQL Interpreter*: It executes SQL queries generated from LLMs.

It is worth noting that our toolkits can be easily expanded through natural language tool function definitions in a plug-and-play manner. MedAssist employs LLMs as autonomous agents in a multi-turn conversation with a code executor, iteratively refining code based on execution feedback until reaching an optimal solution.

Clinicians often pose complex queries that require advanced reasoning across multiple tables and access to a large number of records within a single query. To accurately identify the necessary tables, we first incorporate query-specific medical knowledge into MedAssist to form a detailed understanding of the query under a limited context length. Given a clinical question *q* and reference EHRs ℛ = {*R*_0_*, R*_1_, ⋯}, MedAssist prompts the LLM to produce domain knowledge *B*(*q*) most relevant to *q*, guiding the identification and location of useful references within ℛ.

Following the background assimilation, MedAssist then integrates LLMs and a code executor in a multi-turn conversation for iterative debugging. Initially, MedAssist generates code *C*(*q*) that interacts with the EHR database to extract and process relevant data. The generation of *C*(*q*) draws upon the database introduction ℐ, tool function definitions 𝒯, a set of *K*-shot examples *ℰ*(*q*) the original query *q*, and the contextual background knowledge *B*(*q*):

(2) C(q)=LLM([ℐ;𝒯;ℰ(q);q;B(q)]).

The code executor subsequently extracts and executes *C*(*q*) as *O*(*q*) = EXECUTE(*C*(*q*)). When execution errors or suboptimal outputs occur, the executor provides error feedback, enabling MedAssist to refine the code through continued conversation until achieving accurate query resolution.

### 2.3 Knowledge Summarization with LLMs

By combining knowledge from external sources and internal health records, MedAssist leverages LLMs’ strengths in instruction following [6] and summarization [4] to generate personalized, context-aware patient diagnosis and treatment summaries.

Specifically, the summarization module in MedAssist integrates information from both local EHR data and external medical corpora to generate personalized summaries for each patient. The module structures its input into three key components: (1) external knowledge relevant to the patient’s medical codes or queries, (2) the patient’s medical history and retrieved EHR data, and (3) user-defined instructions specifying the focus of the summary. Using this structured input, LLM is able to generate informative, actionable summaries to assist in understanding diagnoses and treatment plans. For clinicians, the summaries from MedAssist highlight diagnostic insights, potential treatment strategies, and key clinical considerations, ensuring relevance and brevity for effective decision-making.

## 3 Demonstration

### Implementation Details.

The MedAssist demo uses a modular architecture with Python for the backend (FastAPI for APIs and query processing) and React for a user-friendly frontend. The demo utilizes CSV files as internal records to simulate EHRs, which, combined with external knowledge sources, serve as input for the model to generate structured insights and summaries. For deployment, the backend is hosted on AWS Elastic Beanstalk, and the frontend is deployed on AWS S3 with CloudFront for efficient static file hosting and content delivery. We use the HuggingFace to host the retrieval model and use AutoGen 0.2.0 [11] as the interface for communication between the LLM agent and the code executor.

### Case Studies.

**Figure 3:**
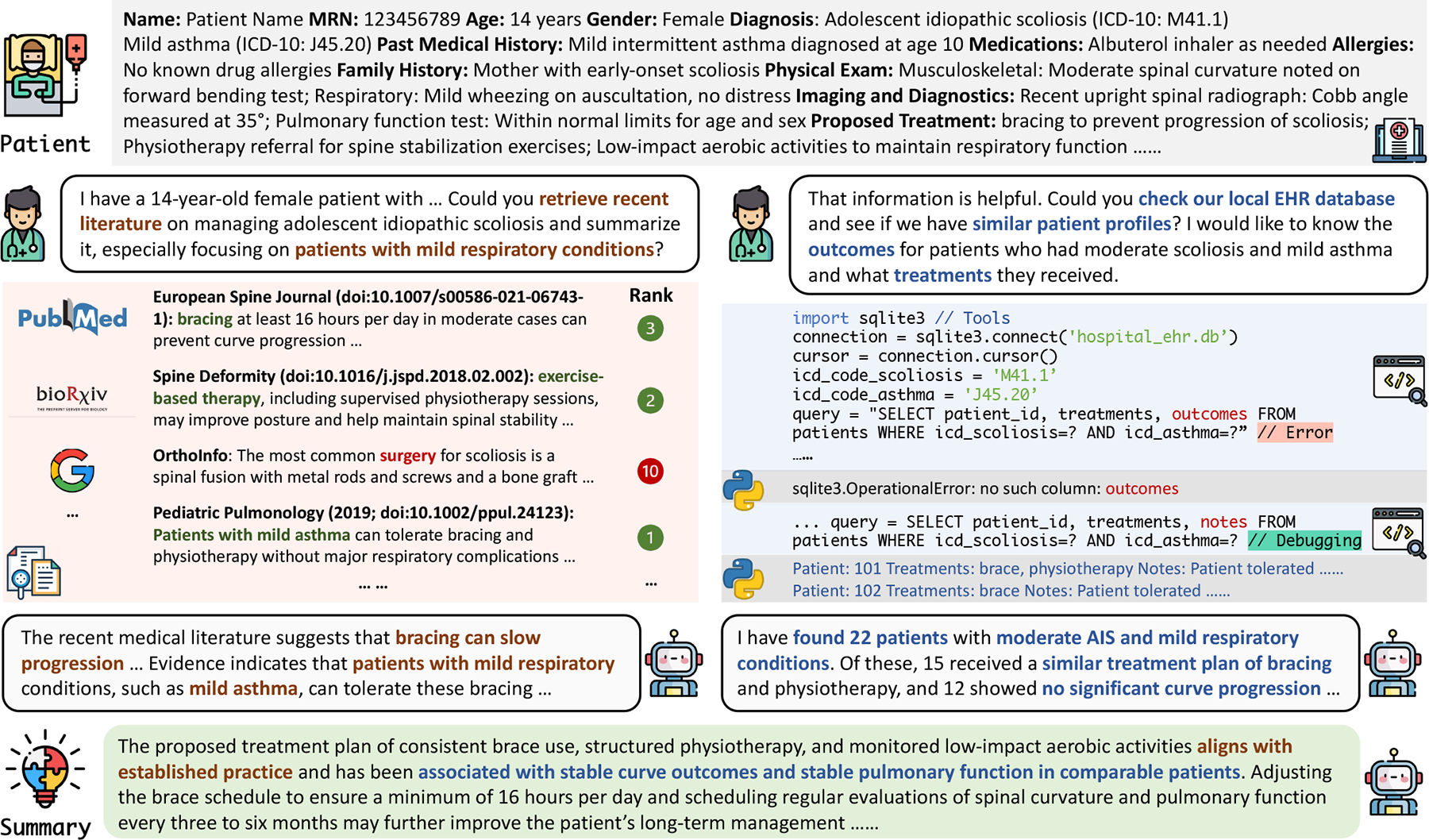
An example of MedAssist on generating informative summaries for a given patient record.

MedAssist provides an interactive system for users to results by leveraging LLMs through the techniques described in Sec. 2. Fig. 3 highlights the system’s three main steps, demonstrated through the case of a 14-year-old patient with adolescent idiopathic scoliosis and mild asthma, illustrated as follows:

- *Knowledge Retrieval*: Given a query for the treatment of with scoliosis and mild respiratory conditions, MedAssist first searches external knowledge sources like PubMed and bioRxiv. It identifies key evidence supporting bracing and physiotherapy as effective treatments without adverse respiratory effects.
- *Data Retrieval*: MedAssist queries the local EHR database to find similar patient profiles, debugging any errors in the process, and retrieves treatments and outcomes from 22 matching cases.
- *Retrieval-Augmented Summary Generation*: Combining this information, MedAssist generates a concise summary emphasizing that the proposed treatment plan aligns with established practices while recommending specific protocol requirements (e.g., at least 16 hours per day) and structured follow-up evaluations.

## 4 Conclusion

In this demonstration, we develop MedAssist, a system for clinicians to easily interact with EHRs. MedAssist reduce the labor of clinicians for creating patient diagnosis and treatment summaries and streamlines the process of integrating patient data with external medical knowledge. This system has the great potential for assisting clinical decision-making by presenting clear and personalized insights. Future work will focus on expanding MedAssist’s capabilities for multi-modal data, such as imaging and genomic data, and ensuring its robustness across diverse patient populations.

## Figures and Tables

**Figure 1:**
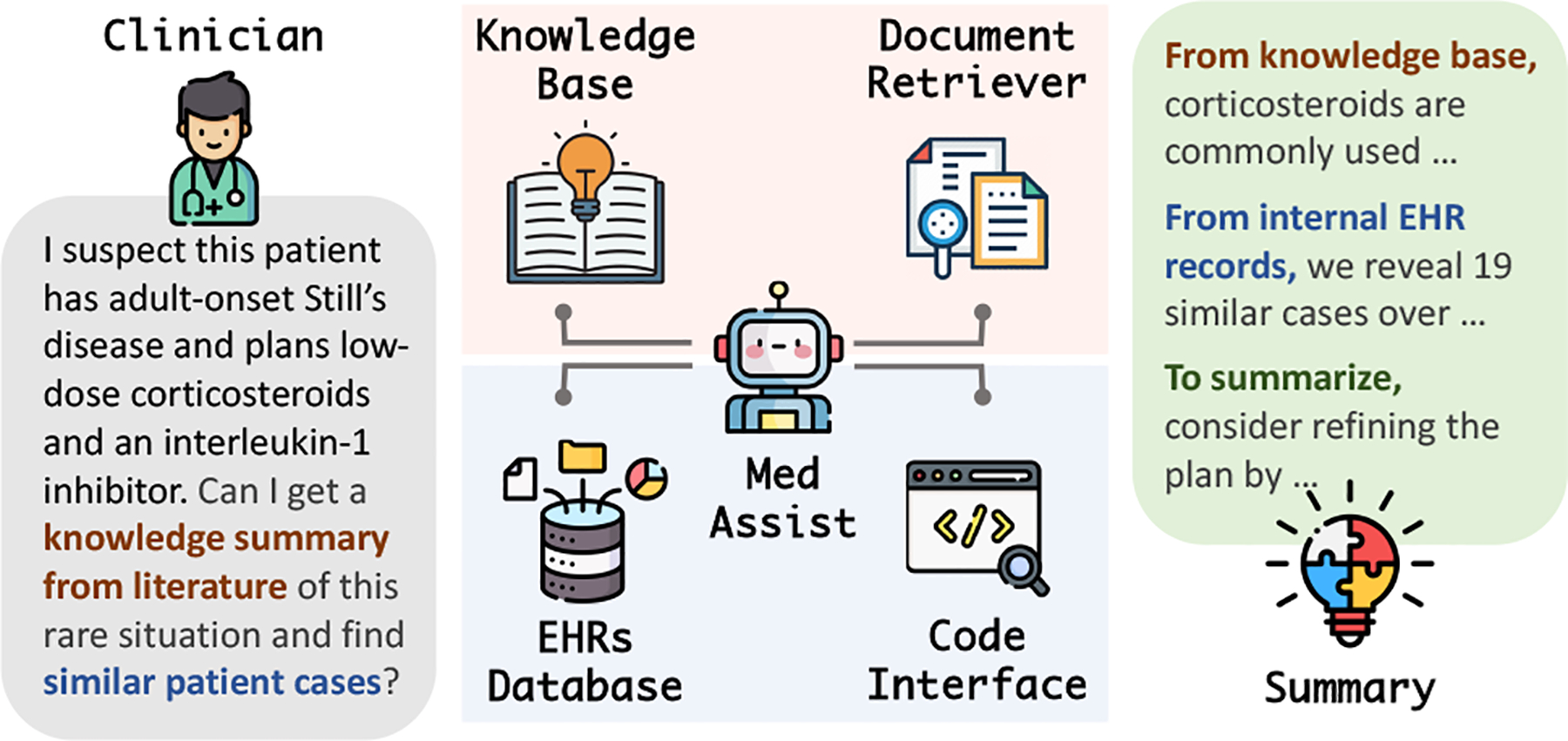
An example of MedAssist. It enables efficient interactions between clinicians and EHR systems and reduce the burden of heavy data engineering with LLMs.

## CCS Concepts

- Computing methodologies → Natural language processing;
- Applied computing → Life and medical sciences.

## Footnotes

1
https://huggingface.co/BAAI/bge-reranker-v2-m3

2
https://products.wolframalpha.com/api
